# On the origin of low‐density neutrophils

**DOI:** 10.1002/JLB.5HR0120-459R

**Published:** 2020-03-14

**Authors:** Marwan Hassani, Pien Hellebrekers, Na Chen, Corneli van Aalst, Suus Bongers, Falco Hietbrink, Leo Koenderman, Nienke Vrisekoop

**Affiliations:** ^1^ Department of Respiratory Medicine and Center for Translational Immunology University Medical Center Utrecht The Netherlands; ^2^ Department of Surgery University Medical Center Utrecht The Netherlands

**Keywords:** granulocytes, LDG, neutrophil subsets

## Abstract

Here we elaborate on the origin of low(er)‐density neutrophils (LDNs) to better understand the variation found in literature. Supplemented with original data, we test the hypothesis that buoyant density of neutrophils is characterized by a spectrum that as a whole shifts to a lower density after activation. Both the 20% highest density (HDNs) and 20% lowest density (LDNs) neutrophils from *healthy* donors were isolated by Percoll of different densities. Using this method we found that LDNs were significantly better in T‐cell suppression and bacterial containment than their 20% highest density counterparts. We found no statistically relevant differences in neutrophil survival or bacterial phagocytosis. Stimulation of healthy donor neutrophils with N‐formyl‐methionyl‐leucyl‐phenylalanine induced LDNs co‐segregating with peripheral blood mononuclear cells after Ficoll separation. These in vitro induced LDNs showed increased activation markers compared to HDNs and were comparable to the activation markers found on the LDN fraction seen in patients with chronic inflammatory conditions such as present in cancer patients. This all fits with the hypothesis that the density of neutrophils is distributed in a spectrum partially coupled to maturation. Additionally a shift in this spectrum can be induced by in vitro stimulation or by activation in disease.

## Graphical abstract

Nowadays the term low(er) density neutrophils (LDN) is used to describe a specific subset of neutrophils with a certain buoyant density, but these LDNs are characterized by a wide variety in both functional and morphologic properties, especially in disease.

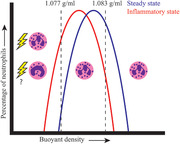

**Abbreviations**
CD: cluster of differentiation
fMLF: N‐formyl‐methionyl‐leucyl‐phenylalanine
HDN: higher density neutrophils
LDN: low(er) density neutrophils
NDN: normal density neutrophils
PAF: platelet activating factor
PBMC: peripheral blood mononuclear cells
SLE: systemic lupus erythematosus

## 1 INTRODUCTION

Neutrophils are main actors in the innate immune system. Until recently neutrophils were thought to belong to a relative homogeneous population of cells. However, an increasing number of studies now show heterogeneity in morphology, phenotype, function, or a combination of these factors.^1^ One of the subtypes of neutrophils identified and studied is the low‐density neutrophil (LDN). It was first recognized in 1986, when neutrophils co‐segregated with mononuclear cells after density‐gradient isolation in systemic lupus erythematosus (SLE) and rheumatoid arthritis patients.^2^ Thereafter, the presence of LDNs have been shown in several, mostly chronic, diseases.^3^

Although the term “LDN” is used nowadays to indicate a distinct neutrophil subset, it actually refers to a wide variety of neutrophils in different pathologic circumstances. In fact, LDNs are poorly defined when it comes to morphology and function. For example, both proinflammatory and anti‐inflammatory properties have been linked to LDNs in inflammatory diseases.^3^ Also, both immature (banded nucleus), as well as hypersegmented neutrophils have been identified in the mononuclear layer.^3^ Interpretation of the above mentioned studies in terms of ontogeny of the LDNs in vivo is hampered by the fact that the onset of inflammatory disease is difficult to determine. It is important to study the kinetics of LDNs to discriminate between activation and a separate lineage. In this hybrid review we have combined existing data, found in the literature, with new original experimental data in order to better understand the origin and functional properties of LDNs in health and disease.

## 2 DENSITY IS A SPECTRUM IN NEUTROPHILS

In 1983, Pember et al. showed that heterogeneity of density with a bell‐shaped distribution exists within the healthy human circulating neutrophil pool.^4^ In addition, in this study density and mean cell volume were found to be connected. A similar distribution of different densities was also found in elicited and nonelicited murine neutrophils, and in neutrophils in allergic human subjects.^5^, ^6^

More recently, Herteman et al. studied LDNs in asthmatic and healthy horses and found that, although their numbers varied between health and disease, the functional characteristics were similar regardless of health status.^7^ So, LDNs in asthmatic and healthy horses both had an increased capacity to produce neutrophil extracellular traps, and were similarly more sensitive to activation by phorbol‐12‐myristate‐13‐acetate.^7^ LDNs in both conditions had more N‐formyl‐methionyl‐leucyl‐phenylalanine (fMLF) receptors and were smaller in size, which according to the authors made them intrinsically different. LDNs were described in several studies in healthy volunteers as well.^8^, ^9^, ^10^

Besides a distribution of buoyant densities, other studies have shown functional heterogeneity within the circulating neutrophil pool of healthy humans.^11^, ^12^, ^13^ For instance, in the studies of Eggleton et al. a similar Gaussian‐shaped profile on the basis of different cell surface electrical charge was found.^12^, ^13^ The presence of heterogeneity in the density and functionality in healthy neutrophils prompted us to test the hypothesis that a spectrum of neutrophil densities associated with different functionalities are already present in the circulation of healthy humans.

**FIGURE 1:**
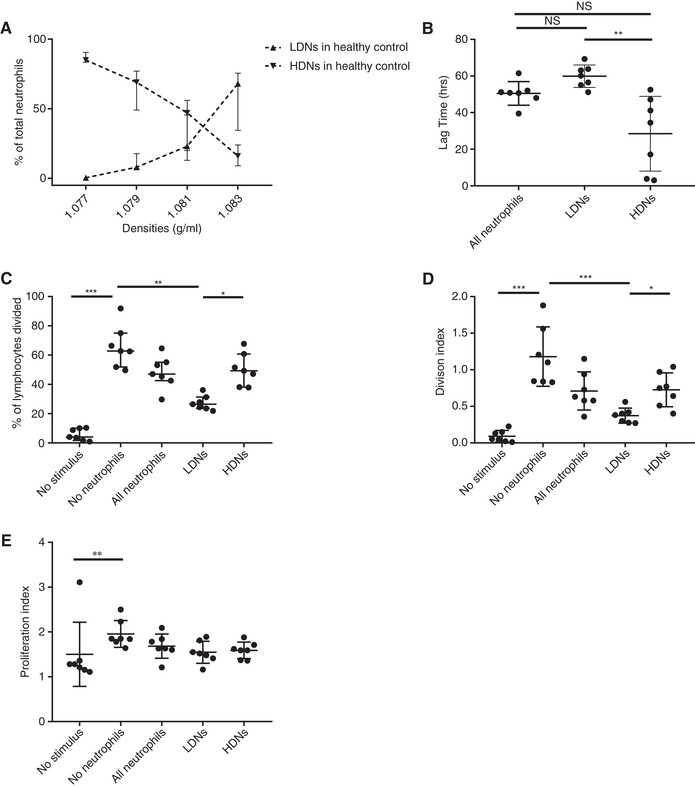
**Unstimulated neutrophils display a spectrum of densities**. Neutrophils were first isolated using Ficoll density centrifugation. Thereafter isolated neutrophils were centrifuged on top of Percoll with different densities. (**A**) Depicted is the percentage of total neutrophils in the “PBMC layer” after density centrifugation with different densities of Percoll (*n* = 9). (**B**) The lag time in which higher density neutrophils (HDNs) and lower density neutrophils (LDNs) contain growth of GFP labeled *S. Aureus* (*n* = 7). (**C**) The percentage of the original population of lymphocytes that divided after stimulation in the absence of neutrophils, in the presence of all neutrophils and in the presence of either LDN or HDN of the same donor (*n* = 7). (**D**) Division index (average number of cell divisions that a cell in the original population has undergone) of lymphocytes under the same conditions as (**C**). (**E**) Proliferation index (the total number of divisions divided by the number of cells that went into division) of lymphocytes under the same conditions as (**C**). For all graphs median ± IQR% is shown. Data are analyzed using Friedman test without correction for multiple comparisons. (**C‐E**) all conditions were tested, but only statistically significant results are indicated

Unstimulated neutrophils from healthy volunteers were centrifuged over different Percoll densities between 1.079 and 1.083 g/ml. Similarly, as in the study by Pember et al.,^4^ the number of cells found in the upper ring increased when higher Percoll densities were used for centrifugation, but not all cells shifted up (Fig. 1A). Some donor variation was present but in all donors more cells stayed in the ring of Percoll with a high density (1.083 g/ml) compared to the Percoll with a low density (1.079 g/ml; Fig. 1A). This proved that a natural spectrum in densities was present in the circulating neutrophil pool, even in healthy individuals. Although neutrophil function is known to change with age,^14^ we did not find a correlation between total numbers of LDNs or HDNs and the donors age (Supporting Information Fig. S1). Next, we tested whether the 10–20% of the lowest density neutrophils (LDNs, typically found on top of Percoll gradient with a density of 1.081 g/ml) had different functional properties compared to the 10–20% of the highest density neutrophils (HDNs, typically found on the bottom of Percoll gradient with a density of 1.083 g/ml). All neutrophils combined were used as a control because they also contain the cells with the intermediate densities.

First, a containment assay was used to compare LDN and HDN functionality, as described by us before.^15^ This assay mimics the in vivo situation and tests several neutrophil functions: chemotaxis, phagocytosis, bacterial killing, and neutrophil survival. The delay in GFP‐labeled bacterial outgrowth (lag time) serves as a measure for the antibacterial capacity of the different neutrophils. There was a clear difference in the containment capacity of *Staphylococcus aureus* of neutrophils in favor of the 20% lowest density neutrophils (Fig. 1B). The extent of the advantage of the LDNs compared to the HDNs varied between the experiments but was always in favor of the LDNs. The containment capacity of the total neutrophil fractions showed a lag time comparable to LDNs. This implies that HDNs are less capable of containing bacteria compared to the other neutrophils in the density spectrum. To rule out the overrepresentation of eosinophils in the 20% highest density fraction, the experiment was repeated after sorting out eosinophils by flow cytometry cell sorting, after which the same defect in bacterial containment capacity was seen (data not shown). Knowing that phagocytosis was comparable between the fractions (Supporting Information Fig. S2A‐C), the enhanced containment of bacteria by LDN was suggested to be an effect of either bacterial killing or neutrophil survival. To differentiate between these possibilities a survival assay was performed, which did not show any difference between the different fractions (Supporting Information Fig. S2D). Therefore, it can be argued that the advantage seen in this bacterial containment assay is due to different capacity in bacterial killing.

Next, we tested putative differences in the capability to suppress proliferation of lymphocytes. It has been previously shown that different neutrophil phenotypes have the capability to suppress lymphocyte proliferation,^16^, ^17^ which has also been linked to LDNs found in a variety of acute and chronic conditions.^18^, ^19^, ^20^, ^21^ Indeed, LDNs isolated from healthy donors, showed a better capability to suppress lymphocyte proliferation after 4 d, especially when they are compared with the HDNs (Fig. 1C‐E). Interestingly, the division index—which takes all cells into account—is clearly different (Fig. 1D), whereas the proliferation index (Fig. 1E)—which only takes the dividing cells into account—is not. It is therefore possible that when lymphocytes have “escaped” the suppression, they do not seem to be sensitive for neutrophil‐mediated suppression anymore. So, from the data mentioned earlier, it can be concluded that a property to suppress proliferation of lymphocytes is linked to the intrinsic buoyant density of neutrophils from healthy donors.

## 3 ACTIVATION OF NEUTROPHILS IN VITRO LEADS TO A SHIFT OF DENSITY

Both human and murine neutrophils change density when activated by endotoxin‐activated serum or fMLF in vitro.^4^ Interestingly, this change in density still resulted in a distribution of densities with a bell shape.^4^ This finding suggests that activation of neutrophils leads to a change of density for all cells, but only neutrophils that are in the lower density of the Gaussian distribution prior to activation will segregate in the peripheral blood mononuclear cell (PBMC) fraction after Ficoll centrifugation.

Other proof that lower density granulocytes are an effect of activation was shown when neutrophils were incubated with *Mycobacterium tuberculosis* in vitro.^22^ Because LDNs were also found in patients infected with *Mycobacterium tuberculosis*, it is very plausible that a direct interaction between neutrophils and the bacterium leads to a decrease in density.^22^

Further complexity is introduced when taking into account that neutrophil density gradually decreases when the processing of neutrophils is delayed for more than 6 h.^23^ This “spontaneous” density shift of neutrophils in vitro is likely to contribute to the wide variety of LDN numbers found between different experimental setups.

**FIGURE 2:**
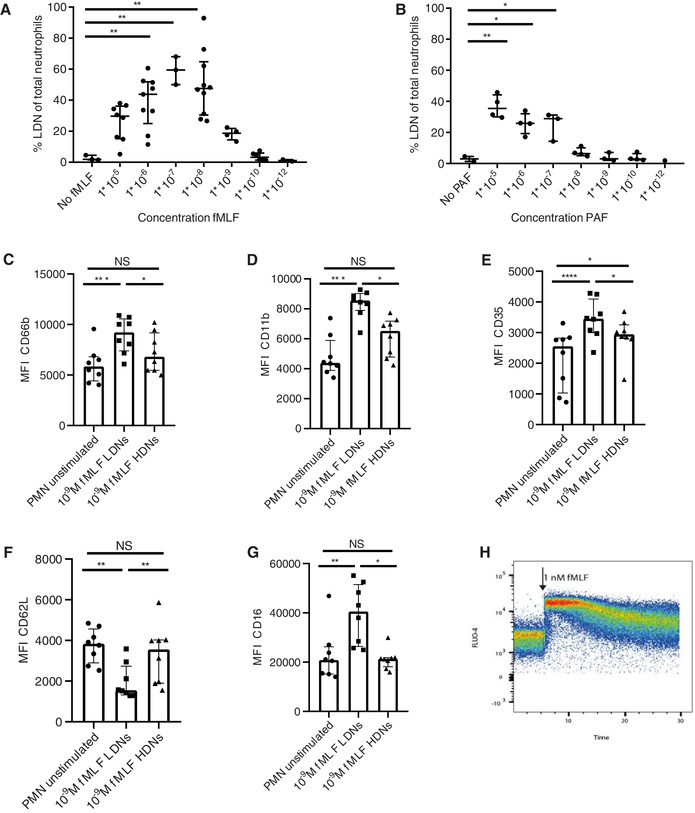
**Density shift of neutrophils after stimulation**. Depicted is the percentage of neutrophils in the PBMC fraction after stimulation of neutrophils with (**A**). N‐formyl‐methionyl‐leucyl‐phenylalanine (fMLF) or (**B**) platelet activating factor (PAF) (**C‐G**) Median fluorescence intensity of activation markers on unstimulated neutrophils and lower density neutrophils (LDNs) and higher density neutrophils (HDNs) after 1 nM fMLF stimulation (*n* = 8). (**C**) CD66b, (**D**) CD11b, (**E**) CD35, (**F**) CD62L, and (**G**) CD16. (**H**) FLUO‐4 signal for gated neutrophils is shown before and after adding 1 nM fMLF. Data are depicted as median with IQR. Data were analyzed using Friedman test without correction for multiple comparison. (**A‐B**) all conditions were tested, but only statistically significant results are indicated

To reproduce that in vitro activation shifts the buoyant spectrum of freshly isolated neutrophils from healthy volunteers, we stimulated neutrophils with fMLF and platelet activating factor (PAF) in a concentration range between 10 μM and 1 pM (Fig. 2A‐B). Indeed, stimulation of neutrophils in vitro caused a decrease in density in at least part of the neutrophils. After fMLF stimulation, the fraction of the neutrophils that was found in the PBMC fraction because of a decreased buoyancy <1.077 g/ml followed a bell‐shaped curve, with the maximum shift at 0.01 μM (Fig. 2A). In contrast, when stimulated with PAF, in the density shift appeared in a concentration dependent manner with the highest concentration of PAF resulting in the highest percentage of LDNs (Fig. 2B). These results complicate the understanding of LDNs even further, because apparently not only the stimulating agent itself is important in the number of LDNs that are generated, but also the concentration of the stimulus.

Next, fMLF‐stimulated neutrophils were analyzed after Ficoll‐Paque density centrifugation, using a multicolor flow cytometry panel. This experiment showed that after 1 nM fMLF stimulation, all neutrophils (LDNs and HDNs) show a change in expression of activation markers (Fig. 2C‐G). However, there was a remarkable difference in activation markers between LDNs and HDNs. LDNs showed a higher expression of CD66b, CD11b, CD35, and CD16 and a lower CD62L expression after 1 nM fMLF stimulation (Fig. 2C‐G). This is in contrast with unstimulated neutrophils of different densities, which did not show a difference in activation markers between LDNs and HDNs (Supporting Information Fig. S3). A higher concentration of fMLF (0.1 μM) also led to an increase of activation markers in both density fractions, but the differences between LDNs and HDNs were less pronounced (Supporting Information Fig. S4). This could mean that during homeostasis, certain neutrophils are more responsive to a lower concentration (1 nM) of fMLF with a subsequent shift in density of only these cells. However, because all neutrophils show a Ca^2+^ influx with this low concentration of fMLF (Fig. 2H), another possible explanation could be that all cells shift in density but, as described in Section 2, only the cells on the lower end of the spectrum end up in the PBMC fraction.

## 4 LDNS IN DISEASE ARE HETEROGENEOUS

The appearance of neutrophils in the PBMC fraction after Ficoll‐Paque centrifugation is described in several inflammatory diseases. However, when different patient groups and different studies are compared, these cells are quite variable in both phenotype and functionality.^3^, ^9^, ^20^, ^24^, ^25^ In the following paragraphs, the different subsets of LDNs are described, supplemented with our own data. A more extensive description of LDN in inflammatory diseases has been summarized recently in an excellent review by Scapini et al.^3^

### 4.1 Are LDNs mainly immature neutrophils?

In the human bone marrow, a progressive increase of buoyant density was described for maturation of cells within the granulocyte lineage.^26^, ^27^ Banded and segmented neutrophils displayed a density above 1.080 g/ml, whereas promyelocytes and myelocytes exhibit a density below 1.080 g/ml.^27^

Both acute and chronic inflammatory conditions often lead to an enhanced (emergency) granulopoiesis in the bone marrow^28^, ^29^ that will subsequently lead to the appearance of immature neutrophils in the circulation, often referred as a left shift.^30^ Similarly, in a model of systemic inflammation, human experimental endotoxemia also leads to the appearance of banded neutrophils in the circulation.^31^ Because neutrophil progenitors have a lower buoyant density, it is not surprising that in certain inflammatory conditions such as cancer or sepsis these immature neutrophils are found in the PBMC fraction.^19^, ^32^, ^33^

**FIGURE 3:**
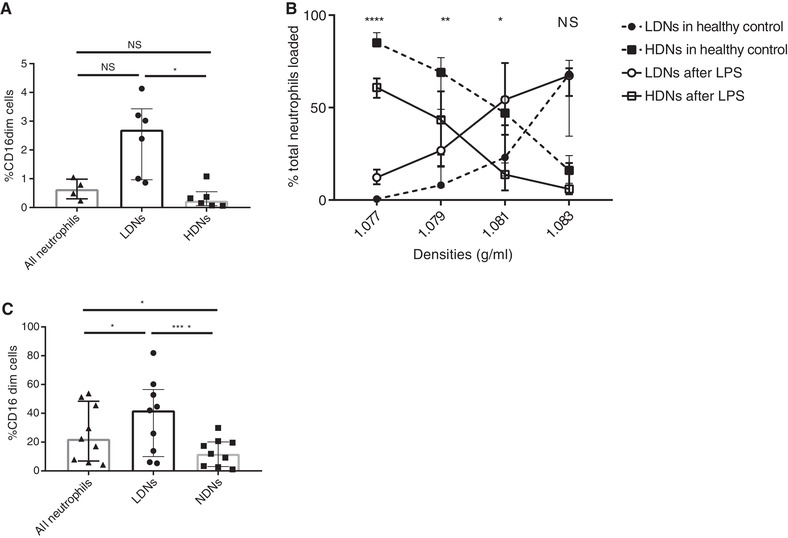
**CD16^dim^ neutrophils (banded nucleus) are more abundant in the lower density neutrophil (LDN) fraction**. (**A**) Depicted is the percentage of CD16^dim^ neutrophils of healthy donors in all neutrophils, LDN and higher density neutrophils (HDN) (*n* = 6). (**B**) Presented is the percentage of the total loaded neutrophils in the PBMC (LDNs) or PMN (HDNs) layer. The dashed lines depict the normal unstimulated situation (*n* = 9, also see Fig. 1A), whereas the solid lines represent the neutrophils of different healthy volunteers after an in vivo LPS challenge (*n* = 8). (**C**) Percentage of CD16^dim^ neutrophils in the LDN and HDN fraction and for all neutrophils after LPS challenge (*n* = 9). Data are depicted as median with IQR. Data of (**A**) and (**C**) were analyzed using Friedman test without correction for multiple comparison. Data of (**B**) was analyzed using a Mann‐Whitney test

To test whether density is linked to maturation of neutrophils in healthy volunteers, we used a multicolor flow cytometry analysis on unstimulated neutrophils after Percoll density centrifugation. Although their numbers were small in healthy donors, a striking difference was found in the amount of CD16^dim^/CD62L^high^ banded neutrophils in the 20% lowest density neutrophils (median of 2.7% in LDNs vs. median of 0.24% in HDNs; Fig. 3A).^34^

Next, we challenged healthy volunteers with LPS (2 ng/kg), as a model of controlled acute systemic inflammation as was described earlier.^35^ After LPS more CD16^dim^/CD62L^high^ banded neutrophils were recruited to the blood. Significantly more neutrophils appeared on top of the lowest density gradient (1.077 g/ml) after LPS compared to the healthy volunteers that is depicted in Figure 1A (see also Fig. 3B). In line with this, more neutrophils appeared on top of the lower Percoll density gradients (1.079 and 1.081 g/ml). After phenotyping the lower and normal density neutrophils, a difference in numbers of CD16^dim^/CD62L^high^ neutrophils between the PBMC and the granulocyte fraction after Ficoll‐Paque centrifugation (median of 42.0% in LDNs vs. 11.9% in NDNs) was again apparent (Fig. 3C). Our findings are in line with previous findings in bacterial sepsis where the majority of neutrophils in the PBMC fraction had a banded nucleus.^32^ Concluding, our findings indicate that both during homeostasis and acute inflammation, more immature (CD16^dim^) neutrophils are found in the lower density fraction of neutrophils. This means that not only (early) progenitors (promyelocytes and myelocytes) have a lower density compared to mature neutrophils, but also banded neutrophils.

### 4.2 Are LDNs mainly activated/degranulated neutrophils?

As was discussed in Section 3, mature neutrophils from healthy individuals decrease their density when activated in vitro, whereas mature neutrophils in inflammatory conditions can already be found in the PBMC fraction at steady state without additional in vitro activation. These inflammatory LDNs often show signs of activation, especially when compared with neutrophils of healthy donors.^6^, ^8^, ^22^, ^36^ LDNs from patients with advanced adenocarcinoma in particular displayed higher expression of activation markers such as CD11b and CD66b compared to the normal density neutrophils from the same patients.^37^ This difference in activation markers between LDNs and NDNs in advanced cancer is remarkably similar to the situation that is present after stimulation of healthy donor neutrophils with 1 nM fMLF (Fig. 2). It is therefore tempting to speculate that during (chronic) inflammation a subset of (mature) neutrophils is more susceptible to activation and that these mature activated neutrophils are the neutrophils found in the PBMC fraction in disease.

It is believed that this activation reflects degranulation in vivo.^3^ However, it cannot be excluded that LDNs are not in vivo activated but rather primed cells that are more susceptible for in vitro manipulation. Arguments for in vivo degranulation are provided by measuring the expression of certain granule markers on the surface of neutrophils, such as CD63 (azurophilic granules), CD66b (specific granules), gelatinase granules (CD11b), and secretory vesicles.^37^, ^38^ The extent of degranulation in vivo is poorly understood as the data of the actual content of the different granules and vesicles in neutrophils, for example, microscopy data, is scarce. Furthermore, existing electron microscopy data of the granular content of LDNs does not show signs of significant degranulation.^39^ Another argument against degranulation as the cause for the change in buoyant density is the fact that the majority of the in vitro activated neutrophils revert to their original density after only a couple of hours (Supporting Information Fig. S5).^5^ It is quite unlikely for neutrophils to regain granular content in this short time frame. An alternative mechanism could be a change in mean cell volume as a result of water uptake, regulated by aquaporin‐9.^40^ Changes to a higher forward scatter and a lower side scatter by flow cytometry, which is an argument often used to provide proof of degranulation, could also be caused by this phenomenon.^24^, ^41^

The activated phenotype of circulatory LDNs is perhaps acquired in the tissue. Although, this has not been directly shown, neutrophils in the tissue have a very similar phenotype compared to in vitro activated neutrophils.^42^, ^43^ This could mean that LDNs are cells that have reverse transmigrated from the endothelial membrane. Moreover, evidence has been provided that homing of neutrophils to the lung tissue is sufficient to adopt an activated phenotype, irrespective of inflammatory disease.^44^

### 4.3 Are LDNs a distinct lineage of cells?

In SLE the presence of LDNs has been established by multiple studies.^25^, ^39^, ^45^, ^46^ These neutrophils are believed to have a contribution to the pathogenesis of SLE by an enhanced capability to produce type I IFNs and release neutrophil extracellular traps.^39^, ^47^ Moreover, it is suggested that these LDNs have an immature phenotype because of a premature release from the bone marrow, instigated by stimuli, which are produced in the course of the disease.^39^

However, other studies have suggested that LDNs in SLE are a complete distinct subset of neutrophils, because they evidently differ from NDNs.^25^, ^39^, ^45^ For example, in the study of Singh et al. an increased level of copy number alterations, losses of heterozygosity and microsatellite instability were found in LDNs and not in NDNs.^45^ This made the authors suggest that LDNs are an abnormally developed lineage of neutrophils as a result of genomic damage.^45^ Although, this is an interesting model, further research is needed to elucidate these findings. Furthermore, it is still unknown if these findings are specific for SLE or that LDNs from other diseases share these characteristics in genetic variations.

## 5 CONCLUDING REMARKS

Here we found that circulating neutrophils in healthy individuals are heterogeneous regarding their buoyant density and that low density correlated with the increased responsiveness to fMLF, increased bacterial containment, and the capacity to suppress T‐cell proliferation. Although it is difficult to compare healthy neutrophils with neutrophils from patients with different inflammatory conditions, it is tempting to speculate that the cells present in the 20% lowest density fraction of our healthy donors are at least in part comparable with the immunomodulatory cells found in the PBMC fraction in inflammatory conditions such as SLE and cancer. Therefore, we believe that buoyant density is not a distinct feature in a subset of neutrophils found in disease, but rather a spectrum of different densities already found in healthy individuals. An inflammatory condition can subsequently lead to a decrease of the buoyant density of all neutrophils but only the cells on the lower end of the spectrum will shift to the PBMC fraction after Ficoll‐Paque density centrifugation (Graphical Abstract).

Neutrophils with immunomodulatory capabilities are accompanied with an intrinsic lower buoyancy in both health and disease. One can only speculate what the underlying mechanism for the spectrum in different buoyant densities is during homeostasis. However, the shift of the spectrum during inflammation can be related to neutrophil maturation stage, activation status, and granulation. We do not exclude, however, that separate neutrophil lineages are being recruited and constitute part of the LDN fraction during inflammation. We encourage further studies to focus on mechanistic features that can explain a spectrum in buoyant densities in neutrophils rather than a subdivision of only two subsets (LDNs and NDNs).

## 6 MATERIAL AND METHODS

### 6.1 Human volunteers

Blood samples were provided by anonymous, healthy volunteers between the ages of 18 and 65 yr, male and female, after given written informed consent in accordance to the Declaration of Helsinki. All experiments were performed in accordance with the relevant guidelines and regulations. This study was approved by the University Medical Center Utrecht (UMCU) ethical review committee (METC).

### 6.2 Experimental endotoxemia model

The human experimental endotoxemia experiment was induced exactly as described before.^35^ In short, a single dose of 2 ng/kg bodyweight LPS (US Standard Reference Endotoxin *Escherichia coli* O:113, lot #94332B1 obtained from the Pharmaceutical Development Section of the National Institutes of Health, Bethesda, MD, USA) was injected systemically in healthy male and female volunteers with an age between 18 and 30 yr at *t* = 0 h. At *t* = 3 h blood was obtained from the same volunteers. The study was approved by the ethics review board of the Radboud University Medical Center and is in compliance with declaration of Helsinki; International Conference on Harmonisation Good Clinical Practice guidelines, and the rulings of the Dutch Medical Research Involving Human Subjects Act. Written informed consent was obtained from all study participants.

### 6.3 Neutrophil isolation

Human blood samples were collected in sodium heparin tubes (Vacuette Greiner bio‐one, Kremsmünster, Austria). First, whole blood was diluted by adding 1:1 PBS supplemented with 0.32% w/v trisodium citrate (both prepared by the UMCU pharmacy) and 10% w/v human serum albumin (Sanquin, Amsterdam, The Netherlands) (PBS2+), after which it was centrifuged over Ficoll‐Paque (Pharmacia, Uppsala, Sweden) for 20 min at 760 ×*g* at 20°C. The PBMC layer was harvested to check for neutrophils. Then, the red blood cells in the pellet were lysed for 15–20 min in isotonic ice‐cold NH_4_Cl solution. Cells were washed twice and after washing the neutrophils were resuspended in PBS2+, and kept on ice until use. Neutrophil counts were measured on an automatic hematology analyzer (CELL‐DYN Emerald, Abbott, IL, USA). Isolation of the neutrophil yielded a cell suspension with >95% neutrophils in all cases.

### 6.4 Isolation of LDNs after neutrophil stimulation

Activation agents used for stimulation of the isolated neutrophils were N‐formylmethionine‐leucyl‐phenylalaline (fMLF) (Sigma‐Aldrich, St. Louis, MO, USA) and platelet activation factor (PAF) (Calbiochem, EMD Chemicals, Inc., San Diego, CA, USA). For the purpose of the experiments described below each reagent was diluted to 10 μM to 1 pM. Neutrophils were stimulated for 15 min in a water bath at 37°C.

Hereafter, the neutrophils were centrifuged over Ficoll‐Paque as described earlier. The neutrophils that shifted to the “PBMC” layer were regarded as LDNs, the neutrophils that remained below were regarded as HDNs.

### 6.5 Isolation of LDNs without neutrophil stimulation

Percoll with different densities was prepared for these experiments by diluting Percoll with a density of 1.128 g/ml (MP Biomedicals, LLC, Solon, OH, USA) with 10× PBS, PBS, 0.32% w/v trisodium citrate (prepared by the UMCU pharmacy) and 10% w/v human serum albumin solution (Sanquin) in such a way that it yielded different calculated densities: 1.079, 1.081, and 1.083 g/ml. Osmolarity and pH were corrected accordingly to 290 mOsm/kgH_2_O and 7.3, respectively. Granulocytes that were previously isolated with Ficoll‐Paque were, thereafter, centrifuged on different densities of Percoll for 20 min at 760 ×*g* at room temperature. Approximately 10–20% of the lowest density neutrophils, typically found on top of Percoll gradient with a density of 1.081 g/ml and approximately 10–20% of the highest density neutrophils, typically found below the Percoll gradient with a density of 1.083 g/ml were used for further experiments. Comparable with the density shifts after stimulation, these cells were similarly named lower density (LDNs) and higher density neutrophils (HDNs), respectively.

### 6.6 Containment assay

A neutrophil containment assay was used modified from Li et al.^48^ and described by van Grinsven et al.^15^ In short, a gel with fibrin scaffolds was formed to mimic a tissue‐like environment. A total of 100 μL of the fibrin gel was made by mixing 50 μL of 5 × 10^5^ neutrophils/ml in Hepes buffer with 50 μL Hepes buffer containing 2 mg/ml fibrinogen (Sigma‐Aldrich) with 40% human pooled serum (Sigma‐Aldrich), 1 U/ml thrombin (Sigma‐Aldrich), and 5 × 10^5^ CFU GFP‐expressing *S. aureus*. This mix was resuspended before solidification in a 96‐well clear bottom polysterene plate (Corning Costar, New York, NY, USA). Neutrophil survival was determined by adding propidium iodide (PI) to separate wells containing neutrophils. The plates were then incubated in 37°C and the fluorescence of the GFP or PI in single wells was measured every 20 min in the Fluostar Optima or Omega plate reader (BMG Technologies, Ortenberg, Germany). Raw data from the experiments were exported to Microsoft Excel and the lag time was determined as described earlier.^15^

### 6.7 Lymphocyte proliferation suppression assay

Unstimulated lymphocytes were isolated from the PBMC fraction after centrifugation over Ficoll‐Paque. Cells were then stained with CFSE (Sigma‐Aldrich) and washed twice with RPMI 1640 (Gibco, Introgen, Breda, The Netherlands). Thereafter, cells were cultured for 96 h in IMDM (Gibco) supplemented with 5% FCS and 1% penicillin/streptomycin (both from Gibco). A total of 100,000 lymphocytes were activated with anti‐0.15 μg/ml CD3 and 0.2 μg/ml CD28 (both from Sanquin) and incubated with either 200,000 LDNs, HDNs, or total neutrophils (as is explained earlier). After 96 h proliferation of lymphocytes was assessed by determining CFSE dilution on a BD Canto II cell analyzer (Becton Dickinson, Mountain View, CA, USA). Flow cytometry data was analyzed using the FlowJo v10 software (FlowJo, LLC, Ashland, OR, USA). The division index, proliferation index, and percentage of divided lymphocytes were automatically calculated by an algorithm incorporated in FlowJo.

### 6.8 Phagocytosis assay

Isolated LDNs and HDNs, and total neutrophils (as a control) were isolated as described earlier. After isolation the neutrophils were stained with CD45‐APC (clone 2D1; BD, San Jose, CA, USA) for HDNs and CD45‐PE‐Cy7 (clone HI30; BD) for LDNs on ice for 20 min in the dark. Hereafter, the cells were washed once and resuspended in 100 μL supplemented Hepes buffer.

Neutrophil suspension with LDNs, HDNs, or a mix with both neutrophil populations in concentration of 5 × 10^6^ neutrophils/mL was prepared in Hepes buffer supplemented with 40% human serum. GFP‐expressing *S. aureus* was then added with a multiplicity of infection of 1 or 2. After gentle resuspension of the mix, it was placed in a shaking incubator (Innova 44, New Brunswick Scientific, Edison, NJ, USA) at 37°C and 180 rpm. After 40 min samples were taken from the suspension and fixed with 1%PFA (in PBS) on ice.

### 6.9 Flowcytometry analysis

Cells were analyzed using BD LSRFortessa cell analyzer (Becton Dickinson) or BD Canto II cell analyzer (Becton Dickinson). One million cells were stained with antibodies for 30 min in a concentration of 40 million cells per milliliter on ice. Cells were washed twice with PBS2+ before analysis. The following antibodies were used: CD35‐PE/ clone E11; CD62L‐PE‐Cy7/clone Dreg56; CD66b‐AF647/clone G10F5 (all from Biolegend, San Diego, CA, USA); CD11b‐APC‐Cy7/clone ICRF44; and CD16‐PB/clone 3G8 (BD). Neutrophils were differentiated from remaining contaminating cells according to specific scatter patterns on forward and side scatter, CD66b and on CD16 expression (see Supporting Information Fig. S6). Ca^2+^ influx experiments were conducted by loading 1 million cells with 1 μM Fluo‐4, AM (Thermo Fisher, Waltham, MA, USA) for 30 min in 37°C. In the phagocytosis assay neutrophils of different densities were distinguished based on their respective CD45 staining. Banded cells were identified as CD16^dim^ and CD62L^high^ (Supporting Information Fig. S6). For all experiments at least 10,000 events were recorded for data analysis.

### 6.10 Statistical analysis

Data was plotted and statistical analysis was preformed using GraphPad Prism (GraphPad Software, La Jolla, CA, USA). Results are expressed by median ± IQR or mean ± se. A Mann‐Whitney test or a Wilcoxon test was used when only two groups were compared. When comparing more than two groups a Friedman with uncorrected Dunn's test was performed. Statistical significance was defined as *P* < 0.05.

## AUTHORSHIP

M. Hassani and P. Hellebrekers, and L. Koenderman and N. Vrisekoop contributed equally to the manuscript. M.H. and P.H. designed and performed experiments with the help of N.C., C.A., and S.B. F.H., N.V., and L.K. supervised experimental design and data analysis. M.H. and P.H. wrote the manuscript. N.V. and L.K. helped to draft and revise the manuscript. All authors read and approved the final manuscript.

## DISCLOSURES

The authors declare no conflicts of interest.

## Supporting information

tableS1

tableS2

tableS3

tableS4

tableS5

tableS6
